# Expression of Concern on ‘MCPIP1 ribonuclease exhibits broad-spectrum antiviral effects through viral RNA binding and degradation’

**DOI:** 10.1093/nar/gkae342

**Published:** 2024-04-25

This is an Expression of Concern regarding: Ren-Jye Lin, Hsu-Ling Chien, Shyr-Yi Lin, Bi-Lan Chang, Han-Pang Yu, Wei-Chun Tang, Yi-Ling Lin, MCPIP1 ribonuclease exhibits broad-spectrum antiviral effects through viral RNA binding and degradation, *Nucleic Acids Research*, Volume 41, Issue 5, 1 March 2013, Pages 3314–3326, https://doi.org/10.1093/nar/gkt019

In January 2024, the Editors were alerted to several issues with the supplementary Figures S2 and S5, as detailed below.

- Figure S2AB: panel A appears identical to panel B
- Figure S2C: panel C Dox(-) and Dox(+) plates appear identical
- Figure S5: panel C MCPIP1 negative JEV/NS1 and DAPI images appear identical to MCPIP1 C306R negative JEV/NS1 and DAPI images. Panel D MCPIP1 negative DAPI image appear identical to MCPIP1 C306R negative DAPI image.

Upon inspection, the Editors also detected an additional issue with Figure S2A, as detailed below:

- Figure S2A: panel MCPIP1 negative JEV/NS1 and DAPI images appear identical to panel MCPIP1 D141N negative JEV/NS1 and DAPI images, but shifted.

The Editors have contacted the authors and are escalating to the institution. In the interim, we advise readers to examine the details of this study with particular care.

**Julian E. Sale, Barry L. Stoddard**

Senior Executive Editors

